# Nasal Epithelial Organoids as Translational Platforms in Inflammatory, Infectious, and Precision Medicine Applications: A Systematic Review

**DOI:** 10.3390/jcm15114016

**Published:** 2026-05-22

**Authors:** Veronica Scocca, Lorenzo Lauda, Riccardo Nocini, Giovanni Dell’Aversana Orabona

**Affiliations:** 1 Maxillofacial Surgery Unit, Department of Neurosciences, Reproductive and Odontostomatological Sciences, University of Naples Federico II, Via Pansini 5, 80131 Naples, Italy; giovanni.dellaversanaorabona@unina.it; 2 Gruppo Otologico, Via Giacomo Morigi, 41, 29121 Piacenza, Italy; lorenzo.lauda@gruppootologico.it; 3 Department of Otolaryngology Head and Neck Surgery, University of Verona, Piazzale L.A. Scuro 10, 37134 Verona, Italy; riccardo.nocini@univr.it

**Keywords:** nasal epithelial organoids, airway epithelium, chronic rhinosinusitis, cystic fibrosis, CFTR, SARS-CoV-2, respiratory infection, air–liquid interface, precision medicine, translational research

## Abstract

**Background/Objectives:** The airway epithelium plays a central role in host defense, inflammatory signaling, and disease progression across infectious, inflammatory, and genetic respiratory disorders. Human nasal epithelial organoids have emerged as accessible and patient-specific in vitro platforms with increasing translational relevance. This systematic review aimed to critically evaluate the current evidence on nasal epithelial organoid models, focusing on donor characteristics, culture methodologies, differentiation strategies, and translational applications. **Methods:** A systematic search of PubMed/MEDLINE, Embase, Scopus, Ovid MEDLINE, and Cochrane Library was conducted for studies published between 1990 and April 2026. The review followed PRISMA guidelines and was structured according to the PICOTS framework. Eligible studies included in vitro experimental investigations using human-derived nasal epithelial organoids in infectious, inflammatory, or precision medicine contexts. Risk of bias was assessed using the QUIN tool. **Results:** Seventeen studies met the inclusion criteria. Applications clustered into three principal domains: infectious disease modeling, inflammatory and epithelial remodeling research, and cystic fibrosis precision medicine. Most studies employed expandable three-dimensional Matrigel-embedded organoids or organoid-derived air–liquid interface systems. Infection-focused studies demonstrated variant-specific viral replication dynamics and epithelial immune responses, while inflammatory models reproduced disease-associated differentiation and remodeling phenotypes. Cystic fibrosis oriented studies showed that organoid swelling and electrophysiological assays correlate with CFTR functional rescue and, in selected cases, clinical response. Methodological heterogeneity across protocols and outcome reporting precluded quantitative synthesis. **Conclusions:** Human nasal epithelial organoids represent versatile translational platforms bridging accessible patient-derived tissue and advanced airway disease modeling. Although variability in culture protocols and functional benchmarks limits standardization, these models hold significant promise for mechanistic investigation, therapeutic stratification, and precision medicine applications.

## 1. Introduction

Chronic respiratory diseases and airway infections remain among the leading causes of global morbidity and mortality, imposing an escalating socioeconomic burden worldwide [1,2]. At the forefront of respiratory defense lies the airway epithelium, a highly specialized and dynamic tissue that integrates barrier integrity, mucociliary clearance, and finely tuned innate immune responses. Beyond functioning as a passive structural interface, the epithelium actively orchestrates host–pathogen interactions, inflammatory signaling cascades, and tissue remodeling processes that critically shape disease onset and progression [3,4,5,6].

Mounting evidence indicates that epithelial dysfunction is not merely a consequence but a central driver of airway pathology, contributing to chronic rhinosinusitis, asthma, chronic obstructive pulmonary disease, cystic fibrosis, and emerging viral infections [7,8,9,10]. However, mechanistic dissection of epithelial biology has historically relied on animal models and immortalized cell lines, both limited by interspecies divergence, altered differentiation states, and incomplete recapitulation of native airway architecture [11,12,13]. These constraints have hindered translational extrapolation and the development of precision therapeutic strategies.

Primary human airway epithelial models have transformed the landscape of respiratory research by preserving donor-specific molecular signatures and functional phenotypes [14,15]. In particular, human nasal epithelial cells (hNECs) offer a unique translational advantage: they are accessible through minimally invasive sampling, amenable to longitudinal collection, and share structural, transcriptomic, and immunological features with lower airway epithelia [16,17,18,19]. This has positioned nasal-derived systems as robust surrogates for bronchial tissue, enabling scalable and patient-specific modeling of airway disease.

Technological advances have further propelled the field. Air–liquid interface (ALI) cultures permit differentiation into pseudostratified mucociliary epithelia with functional barrier properties [20,21,22], while three-dimensional organoid systems recapitulate epithelial self-organization, cellular heterogeneity, and disease-specific phenotypes in a spatially structured context [23,24]. These platforms have been successfully applied to model viral tropism and persistence, inflammatory remodeling, and CFTR-dependent functional rescue, underscoring their relevance for both mechanistic investigation and therapeutic stratification [25,26,27,28].

Despite this rapid expansion, methodological heterogeneity and variable reporting standards complicate interpretation and comparison across studies. A critical synthesis of available evidence is therefore essential to define the current state of nasal epithelial organoid research, identify translational strengths and limitations, and delineate future directions. Accordingly, this systematic review aims to comprehensively evaluate human nasal epithelial organoid models, focusing on donor characteristics, culture methodologies, differentiation strategies, and translational applications across infectious, inflammatory, and precision medicine domains.

## 2. Materials and Methods

The study followed the Preferred Reporting Items for Systematic Reviews and Meta-Analyses (PRISMA) guidelines (Table S1). Since it involved a review of previously published studies, neither ethics approval nor informed consent were required.

The review was prospectively registered in the PROSPERO database (ID: 1363878) during the early phase of the study, prior to completion of data extraction. In light of the exploratory scope and methodological heterogeneity characteristic of preclinical organoid research, the PROSPERO registration was intended to ensure transparency of objectives, eligibility criteria, and methodological framework, rather than to predefine rigid analytic procedures. No additional standalone protocol was developed beyond the registered record.

### 2.1. Search Strategy

The research covered the years 1990–2026 and included Pubmed/MEDLINE, Cochrane Library, Scopus, Ovid MEDLINE, and EMBASE databases. The broad temporal window was intentionally selected to capture potential early precursor technologies; however, all eligible studies were published between 2020 and 2026, reflecting the recent emergence of nasal epithelial organoid platforms. Relevant keywords, phrases and MeSH terms were tailored to meet the specific requirements of each individual database. An example of the search strategy used for PubMed/MEDLINE was: (“nasal organoids”[Title/Abstract] OR “nasal epithelial organoids”[Title/Abstract] OR “nasal epithelial cells”[Title/Abstract] OR “nasal epithelium”[Title/Abstract]) AND (“inflammatory sinonasal disease”[Title/Abstract] OR “chronic rhinosinusitis”[Title/Abstract] OR “CRS”[Title/Abstract] OR “sinonasal inflammation”[Title/Abstract] OR “respiratory infection”[Title/Abstract] OR “viral infection”[Title/Abstract] OR “bacterial infection”[Title/Abstract] OR “host–pathogen interaction”[Title/Abstract] OR “immune response”[Title/Abstract]). Medical Subject Headings (MeSH) terms were incorporated where appropriate, including “Organoids,” “Nasal Mucosa,” “Rhinitis,” “Sinusitis,” and “Respiratory Tract Infections.” The initial search was conducted on 2 April 2026.

An updated search was subsequently performed on 30 April 2026 using expanded terminology to increase sensitivity and capture heterogeneous nomenclature within the field. Additional keywords included “spheroids,” “apical-out organoids,” “airway organoids,” and “HNEC-derived models.” No additional eligible studies were identified following full-text screening.

A manual cross-reference search of the selected articles was subsequently performed using the snowballing method to ensure comprehensive identification of all relevant studies.

### 2.2. Study Selection

This systematic review was conducted according to the PICOTS framework.

***Patients (P)***

The population of interest included human-derived nasal epithelial cells obtained from both healthy donors and patients affected by inflammatory, infectious, or genetically mediated epithelial disorders affecting the sinonasal mucosa. Inflammatory conditions included chronic rhinosinusitis with or without nasal polyps and allergic rhinitis, while infectious conditions comprised viral, bacterial, and fungal sinonasal infections. Both adult and pediatric populations were considered eligible.

***Intervention (I)***

The intervention consisted of the generation and application of nasal epithelial organoids as experimental and translational platforms. Studies evaluating primary three-dimensional nasal epithelial organoid cultures as well as organoid-derived differentiated systems (including air–liquid interface configurations) were included, provided that nasal-specific data were clearly extractable. Experimental models assessing inflammatory stimulation, host–pathogen interactions, infection dynamics, therapeutic testing, drug-response profiling, or advanced molecular analyses such as transcriptomics, proteomics, or gene editing were considered eligible.

***Comparison (C)***

When available, comparisons included organoids derived from diseased versus healthy donors, infected versus non-infected conditions, stimulated versus unstimulated models, treated versus untreated samples, and three-dimensional organoid cultures versus conventional two-dimensional systems. Studies comparing nasal organoids with other airway-derived organoids were included only when nasal-specific data could be clearly extracted.

***Outcomes (O)***

Outcomes were categorized as primary and secondary according to the experimental domain and translational intent of each study.

Primary outcomes included structural and functional epithelial endpoints directly related to model validation and disease-specific investigation. These comprised morphological and phenotypic characterization of the differentiated epithelium (e.g., lumen formation, presence of ciliated, goblet, basal, and ionocyte populations), assessment of epithelial barrier integrity, ciliary activity, and disease-relevant functional metrics such as CFTR activity or pathogen replication dynamics. In infection-focused studies, primary outcomes included viral or bacterial replication kinetics, multiplicity of infection (MOI) when reported, and time-dependent epithelial responses. In inflammatory and remodeling investigations, primary outcomes included epithelial differentiation patterns, extracellular matrix-dependent changes, and modulation of inflammatory signaling pathways.

Secondary outcomes included immune response profiling (e.g., cytokine and chemokine production), transcriptomic or proteomic analyses, therapeutic response assessment, drug responsiveness testing, and comparative benchmarking between different organoid configurations or culture systems. Translational relevance was evaluated based on the capacity of each model to reproduce disease-specific mechanisms, host–pathogen interactions, and treatment responsiveness in a reproducible and biologically coherent manner.

***Timing (T)***

No restrictions were applied regarding experimental timepoints, and both short-term and long-term organoid culture studies were included.

***Study design (S)***

Eligible study designs comprised in vitro experimental studies and translational laboratory investigations involving nasal epithelial organoids.

Conference abstracts, editorials, letters to the editor, book chapters, and narrative reviews were excluded. Studies were also excluded if they did not specifically involve nasal epithelial organoids or if nasal-specific data could not be clearly extracted. Mixed-cohort studies including multiple airway organoid types were considered eligible only when results specific to nasal organoids were separately reported.

### 2.3. Data Collection Process

The search was conducted independently by two investigators (V.S. and G.D.A.O.). References from the identified databases were merged, and duplicates were removed using the reference management software EndNote^®^ 21 (version 21.5). Articles were screened for relevance based on title and abstract, with those deemed appropriately selected for full-text review. Any discrepancies between the screening authors were resolved through discussion until consensus was reached.

Systematic data extraction from the included studies was made using a structured form, with data archived in a customized Excel^®^ (Microsoft Corp, Seattle, WA, USA) spreadsheet. One author (V.S.) independently extracted the relevant study characteristics, including title, authors, year of publication, model type, application, main outcomes assessed, key findings, limitations, translational relevance, donor characteristics, sample collection methods, organoid generation protocol, differentiation protocol (including air–liquid interface when applicable), final epithelial phenotype, multiplicity of infection (MOI), and experimental timepoints. The accuracy of the extracted data was verified by another author (G.D.A.O.).

### 2.4. Data Synthesis and Analysis

Due to the anticipated methodological heterogeneity across studies, including differences in organoid generation protocols, donor characteristics, inflammatory or infectious models, experimental endpoints, and outcome reporting, a primarily qualitative synthesis was performed. The findings of the included studies were systematically summarized according to the predefined extraction domains, including application, biological and functional outcomes, immune response profiling, pathogen interaction dynamics, and translational relevance.

Given the experimental nature of most included studies, variability in multiplicity of infection (MOI), timepoints, differentiation protocols, and phenotypic endpoints was carefully examined and reported descriptively. Subgroup analyses were planned according to disease category (inflammatory versus infectious), organoid culture system (submerged versus air–liquid interface), and translational application (mechanistic modeling versus therapeutic testing), when sufficient data were available.

Risk of bias was assessed using the Quality In Non-randomized Studies (QUIN) tool [29]. The tool was adapted to the context of in vitro experimental studies involving nasal epithelial organoids. Six methodological domains were evaluated: (i) clarity of study objectives; (ii) donor characterization and metadata transparency; (iii) organoid generation and matrix reporting; (iv) validation of epithelial differentiation and phenotype; (v) completeness of functional outcome reporting; and (vi) statistical transparency and reproducibility. Each study was categorized as low, moderate, or high risk of bias based on the overall methodological quality.

## 3. Results

### 3.1. Study Selection

A total of 457 records were identified through database searching (PubMed *n* = 143, Embase *n* = 150, Scopus *n* = 73, Ovid MEDLINE *n* = 87, and Cochrane Library *n* = 4). After removal of 311 duplicate records, 146 unique articles remained for title and abstract screening. Of these, 109 records were excluded based on irrelevance to the study objectives.

Thirty-seven reports were sought for full-text retrieval. Seven reports could not be retrieved, resulting in 30 full-text articles assessed for eligibility. During full-text evaluation, 13 studies were excluded for the following reasons: not organoid-based (*n* = 3), not exclusively nasal-derived (*n* = 3), not published in English (*n* = 1), inappropriate study design (*n* = 3), duplicate cohort (*n* = 1), comment article (*n* = 1), and book series (*n* = 1).

Seventeen studies met the predefined inclusion criteria and were included in the qualitative synthesis.

**Figure 1:**
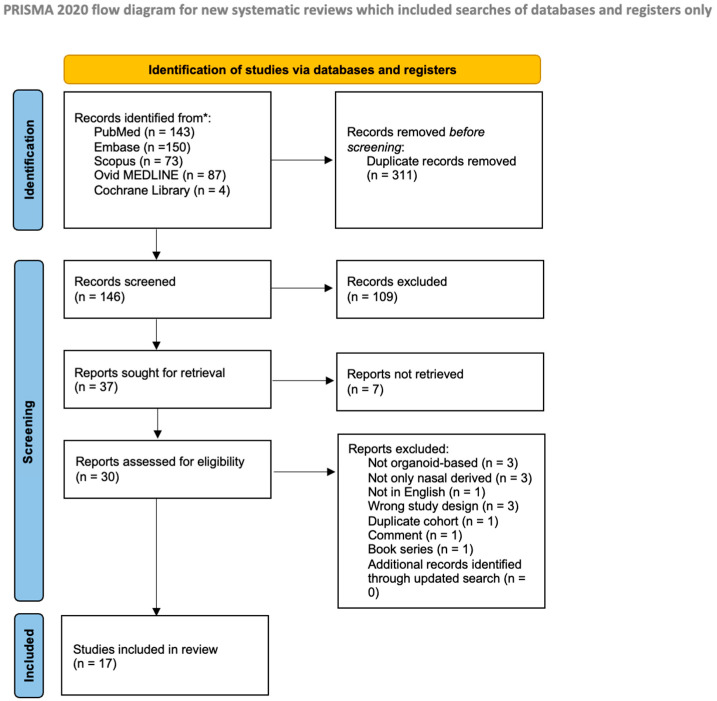
PRISMA flow diagram.

The study selection process is described in Figure 1.

### 3.2. Description of the Studies

The general characteristics of the included studies are summarized in Table 1. To reduce conceptual heterogeneity and facilitate interpretive synthesis, included studies were analytically stratified into three domains: (i) mechanistic infection models, (ii) inflammatory and epithelial remodeling investigations, and (iii) translational precision medicine platforms, primarily focused on CFTR functional assessment. This stratification guided both the qualitative synthesis and the subsequent discussion of methodological maturity.

All included articles were published in English between 2020 and 2026, reflecting the recent and rapidly expanding interest in nasal epithelial organoids as translational research platforms. The majority of studies were experimental in vitro investigations using patient-derived nasal epithelial cells, with a predominance of three-dimensional organoid cultures and air–liquid interface (ALI) differentiation systems.

#### 3.2.1. Mechanistic Infection Models

A substantial proportion of studies focused on infectious disease modeling. Viral infection models were investigated by Aloisio et al. [32,36], Chiu et al. [40], Li C. et al. [38], Wan et al. [34], and Zhu et al. [35], primarily evaluating SARS-CoV-2 and respiratory syncytial virus (RSV) replication dynamics, epithelial immune responses, and variant-specific infectivity. Bacterial colonization and host–microbiota interactions were explored by Boyd et al. [33]. Together, these studies demonstrated the suitability of nasal organoids for modeling upper airway host–pathogen interactions and epithelial inflammatory responses.

#### 3.2.2. Inflammatory and Epithelial Remodeling Investigations

Inflammatory and epithelial remodeling mechanisms were addressed by Ramezanpour et al. [42] and Li L. et al. [37], who investigated chronic rhinosinusitis-derived organoids and extracellular matrix-dependent differentiation pathways, respectively. Ramezanpour et al. [31] further compared different in vitro nasal epithelial culture systems, highlighting the translational relevance of apical-out organoid configurations. These studies emphasized structural and differentiation fidelity rather than pathogen interaction, highlighting matrix composition and polarity configuration as biologically relevant determinants.

#### 3.2.3. Translational Precision Medicine Platforms

Precision medicine and CFTR functional assessment constituted another major area of application. Liu et al. [10,41], Anderson et al. [44], Sette et al. [45], Rodenburg et al. [43], Amatngalim et al. [39], and Borek-Dohalska et al. [30] evaluated CFTR function, drug responsiveness, and theratyping strategies using nasal epithelial organoids. These studies demonstrated the utility of organoid swelling assays, electrophysiological measurements, and genotype-specific drug response profiling as patient-specific biomarkers for therapeutic stratification. Within this domain, functional standardization was more advanced compared to infection and inflammatory studies, with reproducible swelling assays and electrophysiological benchmarking frequently reported.

**Figure 2:**
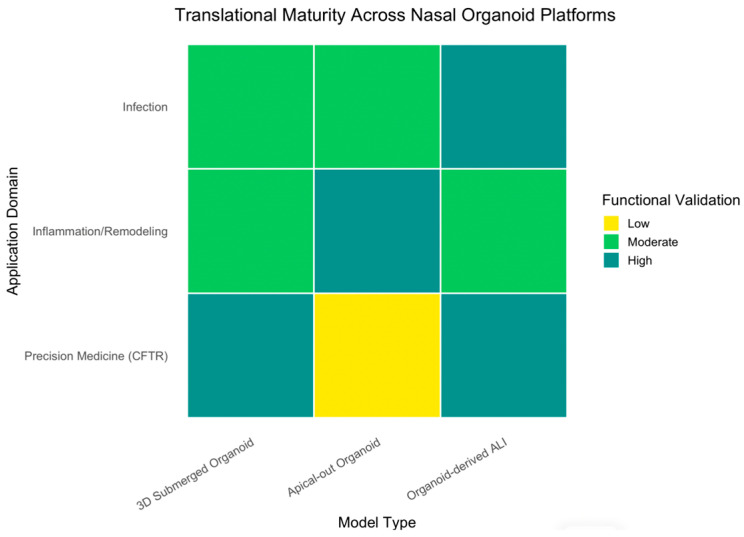
Translational maturity across nasal epithelial organoid configurations. Heatmap summarizing the relative level of functional validation reported across application domains (mechanistic infection models, inflammatory/remodeling investigations, and CFTR-based precision medicine platforms) and principal model configurations (3D submerged organoids, apical-out organoids, and organoid-derived air–liquid interface systems). Functional validation was qualitatively categorized as low, moderate, or high based on the presence of standardized phenotypic benchmarks, reproducible functional assays, and correlation with disease-relevant biological endpoints. The figure illustrates the gradient of methodological maturity identified through qualitative synthesis.

The relative distribution of functional validation across model configurations and application domains is summarized in Figure 2.

### 3.3. Model Type

The included studies demonstrated considerable variability in nasal epithelial organoid model configurations, reflecting the evolving methodological landscape of the field. Most investigations employed patient-derived three-dimensional (3D) organoid cultures generated from nasal brushings or swabs and expanded in extracellular matrix-based systems, predominantly Matrigel. Foundational expandable 3D nasal organoid platforms were described by Liu et al. [10,41], Anderson et al. [44], Rodenburg et al. [43], and Amatngalim et al. [39], which established reproducible lumen-forming structures suitable for functional CFTR assessment and translational drug testing.

Several studies implemented ALI differentiation systems derived from 3D-expanded organoids or directly from stem-cell-enriched epithelial populations. Aloisio et al. [32,36] and Chiu et al. [40] utilized human nasal organoid–ALI (HNO-ALI) platforms to model respiratory viral infections, generating pseudostratified mucociliary epithelia with functional ciliated, goblet, and basal cell populations. Similarly, Boyd et al. [33] employed ALI-differentiated nasal organoids to investigate bacterial colonization dynamics, while Wan et al. [34], and Li C. et al. [38] adapted differentiated organoid monolayers for SARS-CoV-2 infectivity and neutralization assays.

Innovative structural configurations were also reported. Li L. et al. [37] developed apical-out nasal organoids embedded in a collagen–alginate–hyaluronic acid hydrogel system to model extracellular matrix–epithelium interactions, whereas Ramezanpour et al. [31] comparatively evaluated dome organoids, ALI organoids, and monolayer cultures, identifying apical-out ALI organoids as the model most closely resembling in vivo nasal proteomic profiles. Ramezanpour et al. [42] generated expandable chronic rhinosinusitis-derived organoids embedded in Matrigel, providing a disease-specific 3D model.

Comparative airway models were included when nasal-specific data were clearly extractable. Zhu et al. [35] analyzed paired nasal and bronchial organoids derived from the same donors, enabling region-specific immune comparisons. Borek-Dohalska et al. [30] combined nasal epithelial ALI cultures with intestinal organoids for CFTR modulator comparison, though nasal-specific outcomes were distinctly reported.

Overall, the predominant model types comprised (i) expandable 3D Matrigel-embedded nasal organoids, (ii) organoid-derived ALI differentiated monolayers, and (iii) apical-out hydrogel-based systems. Despite methodological heterogeneity in expansion media, differentiation duration, and extracellular matrix composition, the majority of models consistently recapitulated key features of the human mucociliary nasal epithelium, supporting their translational applicability.

### 3.4. Donor Population

The donor populations included in the selected studies were heterogeneous and reflected the broad translational scope of nasal epithelial organoid research. Several investigations utilized healthy adult donors for infection modeling and baseline epithelial characterization [32,33,34,38,40]. These studies primarily focused on viral or bacterial infection dynamics and epithelial immune responses in physiologically differentiated mucociliary models.

Pediatric donors were specifically represented in infection-focused studies. Zhu et al. [35] employed paired nasal and bronchial organoids derived from pediatric subjects, while Aloisio et al. [36] compared infant-derived nasal organoids with those from older donors, demonstrating age-dependent differences in viral susceptibility and inflammatory responses.

Disease-specific cohorts were included in inflammatory and remodeling investigations. Ramezanpour et al. [42] generated organoids from chronic rhinosinusitis (CRS) patients, while Ramezanpour et al. [31] included both CRS patients and non-CRS controls for comparative model evaluation. Li L. et al. [37] established apical-out nasal organoids from surgical specimens of CRS patients to investigate extracellular matrix-dependent differentiation mechanisms.

A substantial proportion of studies [10,30,39,41,43,44,45] focused on cystic fibrosis (CF) populations to assess CFTR function and precision medicine applications, and incorporated genotype-stratified CF cohorts, including F508del homozygous and compound heterozygous mutations, as well as non-CF controls in selected studies.

### 3.5. Organoid Generation

Across the included studies, nasal epithelial organoids were predominantly generated from minimally invasive nasal brushings or swab-derived epithelial cells, followed by stem cell expansion and three-dimensional (3D) culture in extracellular matrix-based systems. Foundational expandable Matrigel-embedded organoid platforms were described by those studies [10,39,41,43,44], in which primary human nasal epithelial cells were expanded—often using conditional reprogramming culture (CRC) or feeder-based systems—and subsequently embedded in high-concentration Matrigel to promote lumen-forming 3D spheroids.

In infection-focused studies [32,33,34,36,38,40], basal epithelial stem cells were expanded and embedded in Matrigel prior to differentiation. These protocols typically involved enzymatic digestion of nasal samples, debris removal, and short-term 3D expansion before downstream differentiation or infection assays. Innovative variations in extracellular matrix composition were introduced by Li L. et al. [37], who developed a collagen I–alginate–hyaluronic acid hydrogel system to generate apical-out nasal organoids, thereby enabling investigation of matrix-dependent epithelial remodeling. Similarly, Ramezanpour et al. [31,42] generated disease-specific organoids from CRS and control donors using Matrigel embedding and comparative expansion strategies.

**Figure 3:**
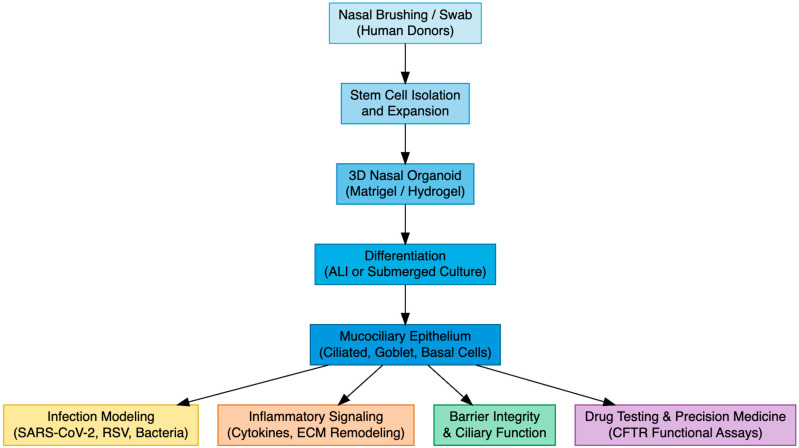
Conceptual Overview of Nasal Epithelial Organoid Generation and Translational Applications.

Overall, although matrix composition, seeding density, and expansion duration varied among studies, most protocols shared a common framework of primary nasal epithelial isolation, stem/progenitor cell enrichment, and 3D embedding to establish expandable organoid cultures (Figure 3).

The methodological and experimental features of the included models are summarized in Table 2.

### 3.6. Differentiation Protocols

Following 3D expansion, differentiation strategies varied depending on the intended translational application. A substantial number of studies [32,33,34,36,38,40] employed ALI differentiation systems to generate pseudostratified mucociliary epithelium. This approach was central to viral and bacterial infection models, where organoids were dissociated into single cells and seeded onto transwell inserts prior to apical air exposure. Differentiation typically occurred over approximately 21 days, resulting in multicellular epithelia containing ciliated, goblet, basal, and secretory cell populations.

In contrast, several CF-focused studies [10,30,39,41,43,44] retained fully 3D submerged organoid configurations for functional assays, particularly forskolin-induced swelling (FIS) analyses. These models demonstrated spontaneous epithelial differentiation within Matrigel, achieving functional maturity typically by day 21.

Alternative differentiation configurations were explored in selected studies. Li L. et al. [37] established apical-out polarity during hydrogel-based differentiation, while Ramezanpour et al. [31] comparatively evaluated dome organoids, ALI monolayers, and apical-out ALI organoids, identifying the latter as most closely resembling in vivo nasal epithelial proteomic profiles. Collectively, despite variability in medium composition, duration, and polarity orientation, the majority of differentiation protocols successfully generated mucociliary epithelial phenotypes suitable for modeling infection, inflammation, barrier function, and precision medicine applications.

### 3.7. Translational Relevance

The included studies collectively demonstrate that nasal epithelial organoids represent a versatile and physiologically relevant translational platform bridging basic epithelial biology and clinically meaningful applications. In the context of infectious disease modeling, organoid-derived air–liquid interface systems were shown to recapitulate upper airway viral tropism, epithelial injury, and strain-specific immune responses. These studies [32,34,35,36,38,40] demonstrated the capacity of nasal organoids to model SARS-CoV-2 and RSV replication dynamics, variant-specific infectivity, cytokine responses, and age-dependent susceptibility. These findings highlight the suitability of nasal organoids as human-relevant alternatives to immortalized cell lines and animal models for studying host–pathogen interactions and evaluating antiviral strategies.

In inflammatory and remodeling contexts [31,37,42] demonstrated that nasal organoids can reproduce disease-associated epithelial phenotypes, extracellular matrix-dependent differentiation processes, and mucociliary functional alterations. These platforms provide mechanistic insight into epithelial barrier dysfunction and remodeling pathways relevant to chronic rhinosinusitis and other inflammatory airway disorders.

Precision medicine applications were particularly evident in cystic fibrosis-focused studies [10,30,39,41,43,44,45], which demonstrated that nasal organoid swelling assays and electrophysiological measurements correlate with CFTR functional rescue and, in selected studies, with clinical treatment response. These findings support the use of nasal organoids as patient-specific theratyping tools and potential surrogate biomarkers for drug responsiveness, particularly in rare genotypes.

Importantly, nasal epithelial organoids are derived from minimally invasive sampling procedures, enabling repeated longitudinal sampling and personalized modeling. While limitations remain—including absence of immune and stromal compartments, donor variability, and protocol heterogeneity—the cumulative evidence supports nasal organoids as scalable, human-derived platforms with strong translational potential across infectious, inflammatory, and precision medicine domains.

### 3.8. Risk of Bias Assessment

Risk of bias was evaluated using the QUIN tool across six methodological domains. Overall, the included studies demonstrated acceptable methodological quality. Most investigations were classified as low risk of bias for clarity of study objectives and completeness of outcome reporting.

Donor characterization showed greater variability, with several studies providing limited demographic or clinical metadata, resulting predominantly in moderate risk assessments in this domain. Organoid generation protocols were generally well described, although differences in reporting depth were observed. Experimental controls and statistical analyses were typically adequate but not uniformly standardized, leading mainly to moderate risk classifications.

**Figure 4:**
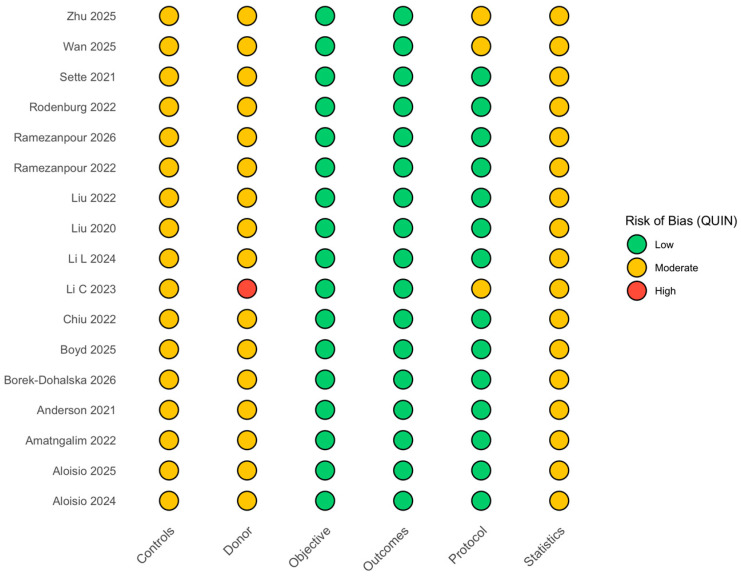
Risk of bias assessment of included studies using the QUIN tool represented as a traffic-light plot across methodological domains.

No study was judged to have consistently high risk of bias across multiple domains. Methodological limitations were largely related to small sample sizes and incomplete reporting rather than fundamental design weaknesses. The detailed domain-level assessment is presented in Figure 4.

## 4. Discussion

This systematic review synthesizes the current landscape of human nasal epithelial organoid and advanced hNEC-derived models, highlighting their rapid transition from descriptive in vitro systems to translationally oriented platforms.

Across applications, a clear gradient of translational maturity emerges. Cystic fibrosis-focused models demonstrate greater methodological standardization and functional benchmarking, whereas infectious and inflammatory applications remain more heterogeneous in terms of differentiation protocols, experimental readouts, and reporting standards. This imbalance has implications for reproducibility and clinical translatability.

A central observation emerging from this review is that nasal epithelial systems have moved beyond their original role as surrogate models of bronchial epithelium. Their accessibility, combined with preservation of donor-specific molecular and functional traits, positions them as uniquely suited for longitudinal sampling and precision-oriented investigation. Within the broader “one airway” framework [14,15], nasal models are no longer merely convenient alternatives but increasingly serve as biologically meaningful proxies in defined mechanistic contexts.

However, the field remains methodologically fragmented. Substantial variability persists in expansion protocols, differentiation timelines, extracellular matrices, and functional assays. Matrix composition (e.g., Matrigel versus defined hydrogel systems), differentiation duration (14 versus 21–28 days), and polarity configuration (apical-in versus apical-out) are not merely technical variables but determinants of epithelial maturation, barrier properties, and functional readouts. Similarly, organoid-derived ALI systems and directly differentiated ALI monolayers may exhibit divergent transcriptomic and proteomic fidelity. These differences significantly influence cross-study comparability and interpretation of translational validity. Such methodological divergence directly affects epithelial phenotype, barrier integrity, and ion transport measurements, thereby influencing translational inference. Importantly, upstream expansion strategy can shape downstream functional readouts, raising concerns regarding cross-study comparability. The absence of standardized quality benchmarks—whether transcriptomic fidelity thresholds, minimal transepithelial resistance values, or defined differentiation criteria—represents a critical barrier to reproducibility [20,24,28]. Even when similar readouts are employed (e.g., FIS assays, cytokine panels, viral replication kinetics), variations in expansion strategies, differentiation duration, and matrix composition may substantially affect outcome magnitude, thereby limiting external validity.

Another important conceptual shift concerns the increasing use of organoid configurations to interrogate epithelial polarity and host–pathogen interactions. Apical-out systems, in particular, reflect a methodological refinement that addresses limitations of traditional 3D cultures. Yet even these advances highlight a persistent limitation: most systems remain epithelial monocultures. While this reductionist approach enables controlled mechanistic interrogation, it does not fully capture epithelial–immune crosstalk, stromal signaling, or vascular contributions that shape airway pathology in vivo. The integration of immune components into co-culture or microfluidic systems will likely represent the next meaningful step toward physiological complexity [22,23].

From a translational perspective, the most mature application currently lies in cystic fibrosis modeling, where functional assays have enabled patient-specific drug testing [44]. In contrast, inflammatory and infectious disease applications, although mechanistically informative, are still limited by variability in infection protocols, multiplicity of infection, and cytokine exposure paradigms. Moreover, many infectious studies rely on limited donor numbers and lack integrated immune or stromal components, warranting cautious interpretation of translational claims. The heterogeneity observed across studies suggests that translational standardization has not yet kept pace with technological innovation.

Donor variability presents a dual-edged phenomenon. On one hand, it introduces experimental noise and complicates reproducibility. On the other, it preserves biologically meaningful interindividual differences that may underlie disease susceptibility and therapeutic response [16,19]. The method of tissue procurement (e.g., brushings, swabs, or surgical specimens) may further influence initial cellular composition and expansion dynamics, yet this variable remains insufficiently analyzed across studies. Future strategies will need to balance standardization with preservation of this intrinsic biological diversity, potentially through structured biobanking initiatives and harmonized metadata reporting.

Finally, structural constraints remain. The reliance on basement membrane extracts introduces batch-dependent variability. Long-term stability of differentiated cultures can be inconsistent. Moreover, the absence of vascularization and systemic influences limits modeling of chronic disease evolution. Addressing these limitations will require integration of defined synthetic matrices, organ-on-chip technologies, and multi-omic profiling approaches capable of validating in vitro fidelity against in vivo benchmarks.

Overall, nasal epithelial organoid systems are transitioning from promising experimental tools to platforms with genuine translational ambition. Yet their future impact will depend less on incremental technological refinement and more on the establishment of standardized methodological frameworks, inter-laboratory validation, and functional benchmarking. The field is now mature enough to demand rigor, not just innovation.

A central question emerging from this review is whether a single “best” methodological configuration can be defined. Our analysis suggests that model suitability is inherently application-dependent. For infectious disease modeling, ALI-differentiated systems appear optimal due to accessible apical surfaces, preserved mucociliary architecture, and reproducible viral entry dynamics. In contrast, submerged 3D organoid systems are particularly suited for CFTR functional assays, where lumen expansion and ion transport readouts provide quantifiable and standardized endpoints. Inflammatory and remodeling studies occupy an intermediate space, where matrix composition, polarity orientation, and epithelial differentiation state critically influence outcome interpretation.

Rather than identifying a universal optimal model, the field may benefit from a stratified methodological framework in which experimental aims guide structural configuration, differentiation strategy, and validation benchmarks. However, such stratification currently lacks standardized criteria and cross-study calibration.

**Figure 5:**
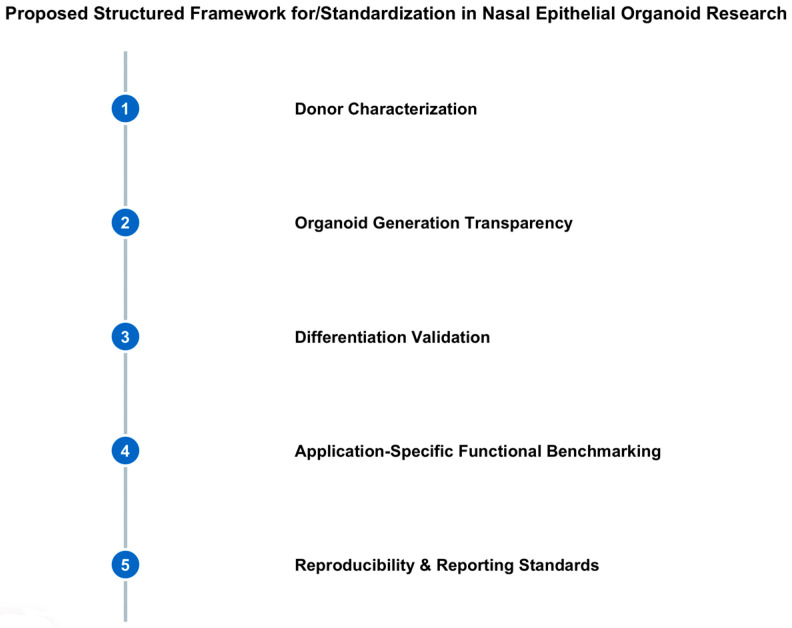
The sequential model outlines five progressive domains required to enhance translational rigor and cross-study comparability.

Building on this perspective, we propose that future nasal epithelial organoid studies adopt a structured quality framework to enhance reproducibility and translational comparability. Essential reporting domains should include: (i) comprehensive donor metadata, including age, disease status, and genotype where applicable; (ii) detailed expansion and matrix composition protocols; (iii) objective validation of epithelial differentiation through ciliation, mucus production, and barrier integrity assays; (iv) standardized functional benchmarking aligned with experimental aims (e.g., MOI normalization in infection studies, FIS calibration in CFTR assays); and (v) transparent statistical and replication strategies (Figure 5).

Such an implementation framework could function either as a reporting checklist or as a benchmarking tool, enabling the field to move from innovation-driven heterogeneity toward structured translational rigor.

### Limitations

This review has several limitations. First, the included studies exhibited substantial methodological heterogeneity in organoid generation, differentiation protocols, and outcome reporting, which precluded quantitative synthesis and meta-analysis. Second, donor metadata were inconsistently reported, limiting assessment of demographic or disease-related variability. Third, most models were based on epithelial monocultures without immune or stromal integration, potentially reducing physiological complexity. Finally, given the rapid evolution of the field, emerging platforms and unpublished protocols may not yet be represented. These limitations should be considered when interpreting the translational generalizability of the findings.

The inability to perform quantitative meta-analysis reflects the intrinsic methodological diversity of this rapidly evolving field rather than a limitation of the review design. In experimental domains characterized by heterogeneous protocols and non-uniform endpoints, rigorous qualitative synthesis and identification of standardization gaps may offer greater translational value than premature statistical aggregation.

## 5. Conclusions

Human nasal epithelial organoids have rapidly evolved into versatile and clinically relevant translational platforms. While current methodological heterogeneity limits cross-study comparability, the field is progressively converging toward greater structural and functional standardization. With implementation of shared quality benchmarks and harmonized reporting practices, nasal organoid systems are positioned to become central tools for mechanistic airway research, therapeutic stratification, and precision medicine.

## Figures and Tables

**Table 1:** Study Characteristics and Translational Applications.

Title	First Author, Year	Model Type	Application	Main Outcomes Assessed	Key Findings	Limitations	Translational Relevance
**Effect of vanzacaftor/tezacaftor/ivacaftor on cystic fibrosis nasal epithelial cells and intestinal organoids compared to elexacaftor/tezacaftor/ivacaftor**	**Borek-Dohalska L, 2026** [30]	HNO-ALI + PDIOs	Comparison of CFTR functional rescue by ELX/TEZ/IVA vs. VAN/TEZ/IVA using electrophysiology and swelling assays	Short-circuit current (ΔCFTRinh-172 ISC); CFTR activity (% WT); Forskolin-Induced Swelling (AUC) in PDIOs	VAN/TEZ/IVA restored CFTR function to 67% of WT vs. 45% with ELX/TEZ/IVA in HNECs; no significant difference detected in PDIO swelling assays	Small cohort; genotype-restricted (F508del/F508del only); no immune components; in vitro model limitations	Demonstrates superior discriminatory power of nasal epithelial electrophysiology over PDIO swelling for comparing highly effective CFTR modulators
**Nasal organoids as optimal models for studying structure and function of primary nasal epithelial cell cultures**	**Ramezanpour M, 2026** [31]	Primary human nasal epithelial cells (HNECs); comparison of: monolayer, ALI culture, Dome organoids (3D), ALI organoids (3D apical-out)	Comparative proteomics (DIA-MS); ultrastructure (TEM); mucociliary function (CBF); evaluation of best in vitro model reflecting in vivo nasal epithelium	Proteomic profiling (1640 proteins identified); differential expression analysis; GO enrichment (GSEA); ultrastructure (TEM); cilia beat frequency (CBF)	Organoids show fewer perturbed proteomic pathways vs. monolayer/ALI. ALI organoids most closely reflect in vivo proteome. ALI organoids show consistent apical cilia and stable CBF (~3.5 Hz). Dome organoids show internal cilia and more variable CBF (~5.8 Hz; *p* = 0.071 vs. ALI).	Small cohort (*n* = 8); donor variability; contamination from non-HNEC proteins in brushings; no transcriptomic validation	ALI organoids represent a robust model for CRS research, mucociliary function assessment, disease modeling, and drug screening
**Strain-Specific Variability in Viral Kinetics, Cytokine Response, and Cellular Damage in Air–Liquid Cultures of Human Nasal Organoids After Infection with SARS-CoV-2**	**Alosio GM, 2025** [32]	HNO-ALI	Viral infection studies SARS-CoV-2 (WA1, B.1.2, Alpha, Beta, Delta, Omicron)	Viral gene copy number (qRT-PCR); 24-plex cytokine/chemokine panel (Luminex); immunofluorescence (ciliated, goblet, basal cells); ciliary area quantification; epithelial morphology (H&E)	Strain-specific differences in viral kinetics and cytokine response; Delta variant showed delayed viral replication and dampened innate immune activation; all variants caused ciliary damage (Delta less severe at early timepoints); basal cell expansion observed during infection	Small number of donor lines; absence of immune cells; model limited to upper airway epithelium	Demonstrates suitability of nasal organoids as an ex vivo human infection model; useful for variant-specific pathogenicity assessment and epithelial immune response studies
**Nasal microbionts differentially colonize and elicit cytokines in human nasal epithelial organoids**	**Boyd AI, 2025** [33]	HNO-ALI	Bacterial monocolonization model: *Staphylococcus aureus* (USA300), *Streptococcus pneumoniae*, *Dolosigranulum pigrum*	CFU quantification; LDH cytotoxicity; apical/basal cytokines (IL-1α, IL-1β, IL-6, IL-8, CXCL10, CXCL11, etc.); localization to mucus layer	Species-specific colonization kinetics; minimal cytotoxicity; live *S. aureus* induced IL-1 family cytokines (inflammasome signature); *D. pigrum* reduced CXCL10; *S. pneumoniae* increased CXCL11	Single strain per species; only adult-derived HNOs; no immune cells; short-term colonization (≤48 h)	Novel physiologically relevant model for nasal colonization; enables study of epithelial–microbiota dynamics; bridge between in vitro cell lines and animal models
**Organoid-based neutralization assays reveal a distinctive profile of SARS-CoV-2 antibodies and recapitulate the real-world efficacy**	**Wan Z, 2025** [34]	3D patient-derived airway organoids	Organoid-based neutralization assays for monoclonal antibodies (mAbs) SARS-CoV-2 (including Omicron variants)	Neutralization potency of mAbs; viral infectivity; ACE2/TMPRSS2 relevance	Nasal organoids better predict real-world mAb efficacy vs. cell lines; ACE2 low and TMPRSS2 high expression improve biological relevance	No Fc-effector functions captured; slower growth vs. cell lines	Superior preclinical platform for antibody efficacy testing and antiviral development
**Comparison of characteristics and immune responses between paired human nasal and bronchial epithelial organoids**	**Zhu L, 2025** [35]	3D patient-derived airway organoids	Comparative analysis of upper (nasal) vs. lower (bronchial) airway immune responses; RSV host–pathogen interaction model	Ciliary Beat Frequency Whole Exome Sequencing (SNV overlap) Bulk RNA-seq (baseline & post-RSV) RSV replication (ddPCR) Cytokine profiling	>99% SNV overlap between NO and BO 95% transcriptomic overlap NO showed lower baseline immune pathway activation RSV replication higher in NO NO exhibited increased pro-inflammatory cytokines (IL-6, IL-11, RANTES, MIG) BO showed higher IL-10 and anti-inflammatory response	Small sample size (n = 4); Pediatric population only; In vitro model; No in vivo validation; No post-infection ciliary beat frequency measurement	Demonstrates that nasal organoids can serve as a surrogate airway model, but regional immune differences must be considered in translational respiratory research
**Pediatric human nose organoids demonstrate greater susceptibility, epithelial responses, and cytotoxicity than adults during RSV infection**	**Aloisio GM, 2024** [36]	HNO-ALI	Viral infection studies Respiratory Syncytial Virus (RSV)	Viral replication kinetics; epithelial innate immune response (cytokine/chemokine profiling); cytotoxicity assays; epithelial injury and cell composition analysis	Infant-derived HNO-ALIs showed increased viral susceptibility, enhanced epithelial inflammatory responses, and greater cytotoxicity compared to older donor-derived lines	Limited number of donor lines; absence of immune cell components; in vitro upper airway model only	Supports age-dependent differences in RSV susceptibility; useful platform for studying pediatric viral pathogenesis and testing antiviral strategies
**Human apical-out nasal organoids reveal an essential role of matrix metalloproteinases in airway epithelial differentiation**	**Li L, 2024** [37]	Primary HNE progenitor cell-derived 3D apical-out nasal organoids cultured in CAH acid hydrogel system	Study of ECM remodeling in airway epithelial differentiation. Investigation of MMP-dependent regulation of ciliogenesis and goblet cell differentiation.	Establishment of CAH gel-based apical-out human nasal organoids (hANOs); ECM degradation dynamics during differentiation; Expression and activity of MMPs (MMP7, MMP9, MMP10, MMP13); Impact of MMP inhibition on epithelial differentiation; Effects on ciliogenesis and goblet cell differentiation; Comparison with conventional ALI and Matrigel-based apical-in organoids	Successful generation of reproducible apical-out nasal organoids from 20 donors; Progressive upregulation of epithelial-derived MMP7, MMP9, MMP10, MMP13 during differentiation; ECM (collagen I) degradation required for apical-out polarity establishment; MMP inhibition suppresses normal ciliogenesis; MMP inhibition increases goblet cell proportion (goblet cell hyperplasia phenotype); MMP9 inhibition specifically associated with increased goblet differentiation; System better mimics ECM–epithelium interactions compared to Matrigel models	Mechanistic pathways linking MMP inhibition to goblet cell differentiation not fully elucidated; No in vivo validation (e.g., epithelial-specific knockout models); Does not comprehensively evaluate other ECM proteases (e.g., ADAMTs); Primarily focused on differentiation biology rather than disease modeling	Provides a physiologically relevant apical-out nasal organoid model Useful for studying airway epithelial remodeling in inflammatory airway diseases; Potential platform to investigate mechanisms underlying goblet cell metaplasia; Enables modeling of ECM–epithelium interactions in health and disease; May support future therapeutic targeting of MMP pathways in airway remodeling disorders
**Human airway and nasal organoids reveal escalating replicative fitness of SARS-CoV-2 emerging variants**	**Li C, 2023** [38]	3D nasal organoids (NsO), airway organoids, alveolar organoids; differentiated 3D organoids and organoid monolayers	Modeling SARS-CoV-2 variant replicative fitness Entry efficiency Syncytium formation Upper vs. lower airway tropism SARS-CoV-2 WT (HKU-001a) Omicron (B.1.1.529) BA.4 BA.5 BA.2.12.1	Viral replication kinetics in nasal, airway, and alveolar organoid monolayers; Comparative infectivity of SARS-CoV-2 variants (WT, Omicron, BA.4, BA.5, BA.2.12.1); Viral entry efficiency; Syncytium formation and fusogenic activity; Effect of TMPRSS2 and Cathepsin L inhibition on viral growth	Progressive increase in replicative fitness across emerging variants; BA.5 showed enhanced entry efficiency and fusogenic activity in nasal and airway organoids; BA.5 demonstrated attenuated replication in alveolar organoids; Viral spread in upper airway organoids associated with syncytium formation; Evidence of adaptation toward upper respiratory tract epithelium	Number of donors not specified; Differentiation protocol not detailed (referenced previous work); In vitro organoid model (does not fully recapitulate immune system interactions); Limited mechanistic dissection beyond entry and replication assays	Provides physiologically relevant human upper airway model for studying SARS-CoV-2 evolution; Supports concept of viral adaptation toward enhanced transmissibility; Useful platform for testing emerging variants and antiviral strategies; Potential tool for future personalized respiratory infection modeling
**Measuring cystic fibrosis drug responses in organoids derived from 2D differentiated nasal epithelia**	**Amatngalim GD, 2022** [39]	HNOs derived from 2D differentiated HNE	CFTR functional testing using Forskolin-Induced Swelling assay; evaluation of CFTR modulator response (including VX-661/VX-445/VX-770)	CFTR function via organoid swelling; genotype-specific drug response; epithelial differentiation markers (β-tubulin IV, MUC5AC, p63, KRT5)	Organoids derived from uniformly differentiated 2D ALI cultures improved consistency and scalability of CFTR functional assays; enabled reliable detection of genotype-specific responses to CFTR modulators	In vitro epithelial model without immune/stromal components; focused exclusively on CFTR functional assessment	Demonstrates scalable, patient-specific nasal organoid platform for precision drug testing in cystic fibrosis
**Human Nasal Organoids Model SARS-CoV-2 Upper Respiratory Infection and Recapitulate the Differential Infectivity of Emerging Variants**	**Chiu MC, 2022** [40]	HNO-ALI model	SARS-CoV-2 infection modeling and variant-specific infectivity analysis in differentiated nasal organoid monolayers	Viral replication (qRT-PCR); immunofluorescence for viral proteins; ciliary damage; interferon and cytokine responses	Robust apical viral replication; infection predominantly in ciliated cells; delayed interferon response; epithelial damage and loss of cilia consistent with in vivo upper airway infection	In vitro epithelial-only model; absence of immune cells; limited donor variability	Validates HNO-ALI as physiologically relevant model of early upper respiratory SARS-CoV-2 infection; suitable for antiviral and pathogenesis studies
**Culture and Imaging of Human Nasal Epithelial Organoids**	**Liu Z, 2022** [41]	Primary patient-derived 3D HNE organoids (Matrigel-embedded)	Quantification of Baseline Luminal Ratio; Forskolin-Induced Swelling assay; CFTR functional assessment; Imaging-based phenotyping; Preclinical evaluation of CFTR modulators; Platform for gene therapy testing	Total Surface Area (TSA) Luminal Area (LA) BLR (LA/TSA) FIS response (1 h and 8 h) Ciliary beat frequency (µOCT optional) Immunofluorescence markers	Organoids reproducibly form lumen and differentiated airway epithelium BLR correlates with CFTR function 8 h FIS discriminates CFTR functional differences Amenable to automated imaging and high-content analysis	Protocol paper (not powered clinical cohort) Manual luminal measurement described Apical surface not easily accessible No immune component	Minimally invasive sampling Scalable and reproducible platform Suitable for precision medicine and CFTR theratyping Alternative to intestinal organoids
**Characterization of human nasal organoids from chronic rhinosinusitis patients**	**Ramezanpour M, 2022** [42]	Primary patient-derived 3D HNE organoids (Matrigel-embedded)	Morphological and molecular characterization; platform for phenotypic high-throughput screening and drug response research in CRS	Organoid morphology and growth; lumen formation; stem cell marker (LGR5); differentiation markers (MUC2, MUC5, Tubulin, ZO-1, E-cadherin); protein expression (WB); FACS; ciliary beat frequency	Successful generation of expandable CRS-derived nasal organoids; stem cell enrichment (↑ LGR5 vs. monolayer); progressive differentiation with functional cilia; cryopreservation feasible	Only CRS patients (no healthy control comparison); no functional inflammatory or infection modeling; relatively small donor number	Provides a physiologically relevant CRS organoid platform suitable for drug screening, host–pathogen interaction studies, and personalized approaches
**Drug Repurposing for Cystic Fibrosis: Identification of Drugs That Induce 3TR-Independent Fluid Secretion in Nasal Organoids**	**Rodenburg LW, 2022** [43]	Primary human nasal epithelial organoids derived from minimally invasive nasal brushings	Medium-throughput screening of ~1400 FDA-approved drugs to identify CFTR-independent fluid secretion in CF nasal organoids	Organoid swelling (fluid secretion rate/AUC) Plate-normalized swell rate (384-well screening) Validation swelling in CFTR-null donors Chloride conductance (Ussing chamber, Isc) TMEM16A activity (KO validation, YFP assay)	12 FDA-approved drugs induced CFTR-independent fluid secretion, independently of CFTR and TMEM16A	In vitro study only Small donor number Mechanism not fully elucidated No in vivo validation Short-term endpoint (2–3 h)	Primary human CF nasal organoids Minimally invasive sampling Drug repurposing potential CFTR-mutation independent approach No clinical validation yet
**CFTR function and clinical response to modulators parallel nasal epithelial** **organoid swelling**	**Anderson JD, 2021** [44]	HNE organoid Swelling Model	CFTR functional testing and correlation with clinical response	Organoid swelling (AUC); correlation with short-circuit current (Isc); correlation with clinical outcomes (Δsweat chloride, ΔppFEV1)	Organoid swelling correlated with CFTR activity in monolayers and with clinical response to CFTR modulators; distinguished incremental responses to different drug combinations	Small sample size; retrospective clinical correlation; no immune/stromal components	Demonstrates nasal organoids as a patient-specific biomarker for precision modulator therapy selection and potential clinical trial surrogate endpoint
**Theratyping cystic fibrosis in vitro in ALI culture and organoid models generated from patient-derived nasal epithelial conditionally reprogrammed stem cells**	**Sette G, 2021** [45]	Patient-derived HNE CRC-expanded airway epithelial stem cells	Theratyping Testing CFTR modulators (Ivacaftor, Lumacaftor, Tezacaftor, Elexacaftor) Evaluation of Trikafta efficacy in rare genotypes	CFTR protein maturation (band C, immunoblot) Forskolin-induced swelling (FIS) Fluid re-absorption assay (ALI) CFTR mRNA expression	Trikafta showed highest CFTR rescue Similar response in F508del/F508del and F508del/ins genotypes CRC models reliable for personalized theratyping	In vitro model Limited number of rare genotypes tested No direct clinical outcome correlation	Supports personalized CF treatment selection Potential FDA-aligned theratyping strategy Enables testing of rare/orphan genotypes
**Human Nasal Epithelial Organoids for Therapeutic Development in Cystic Fibrosis**	**Liu Z, 2020** [10]	Patient-derived 3D HNE organoid model	Functional assessment of CFTR activity Biomarker of CFTR dysfunction Evaluation of CFTR rescue (CFTR modulators/gene-based therapies) Preclinical testing platform Ex vivo surrogate biomarker for clinical trials Assessment of mucus production, ciliary function, and airway epithelial differentiation	Morphology and differentiation of HNE organoids (lumen formation, epithelial structure, cilia, mucins, tight junctions, CFTR, ionocytes) TSA and LA BLR Correlation between BLR and short-circuit current (ΔIsc) FIS assay (1 h vs. 8 h measurements) Ciliary beat frequency (µOCT) Expression of MUC5AC, MUC5B, ZO-1, CFTR, and FOXI1	HNE organoids recapitulate differentiated human airway epithelium, including functional cilia, mucus production, tight junctions, CFTR expression, and ionocytes. Significant differences in lumen size were observed among: Non-CF subjects, CF patients with residual function mutations, CF patients with minimal function mutations BLR distinguishes non-CF from CF organoids and differentiates levels of CFTR dysfunction. Strong correlation between BLR and baseline forskolin-stimulated short-circuit current (r = 0.94, *p* = 0.0005). The 8 h FIS assay significantly distinguishes CFTR functional differences, whereas the 1 h assay does not. Cultures were reproducible regardless of genotype. The model requires few starting cells but allows expansion and multiple replicates. Automated imaging is feasible for functional assessment.	Functional analyses were performed on a limited number of subjects. Baseline luminal ratio measurements were manually performed (not fully automated). The apical surface is not easily accessible. Further validation in larger patient cohorts is needed. Extended FIS protocol requires additional confirmation.	Ex vivo model derived from nasal brush biopsy (minimally invasive and repeatable procedure). Functional biomarker to evaluate: CFTR correction Gene therapy approaches RNA- and DNA-based systemic therapies CFTR modulators Potential surrogate biomarker in clinical trials for gene therapy. Practical alternative to intestinal organoids (no rectal biopsy required). Suitable for preclinical drug testing and longitudinal monitoring of genetic repair stability.

Abbreviations: ALI, air–liquid interface; BLR, baseline luminal ratio; CAH, collagen–alginate–hyaluronic acid hydrogel; CBF, ciliary beat frequency; CFTR, cystic fibrosis transmembrane conductance regulator; CRC, conditional reprogramming culture; DIA-MS, data-independent acquisition mass spectrometry; FACS, fluorescence-activated cell sorting; FIS, forskolin-induced swelling; GSEA, gene set enrichment analysis; HNE, human nasal epithelium; HNECs, human nasal epithelial cells; HNO-ALI, human nasal organoid–air–liquid interface; Isc, short-circuit current; LA, luminal area; NsO, nasal organoids; PDIOs, patient-derived intestinal organoids; qRT-PCR, quantitative reverse transcription PCR; RSV, respiratory syncytial virus; SNV, single nucleotide variant; TEM, transmission electron microscopy; TSA, total surface area; WT, wild type; µOCT, micro-optical coherence tomography.

**Table 2:** Methodological and Experimental Features of Nasal Organoid Models.

First Author, Year	Model Type	Donors	Sample Collection	Organoid Generation	ALI Differentiation Protocol	Final Phenotype	MOI	Timepoints
**Borek-Dohalska L, 2026** [30]	HNO-ALI + PDIOs	F508del/F508del CF patients (12); healthy WT donors (10) (HNE); WT donor (1) (intestinal organoids)	Nasal brushing Intestinal biopsy-derived organoids	HNE cells expanded in PneumaCult-Ex Plus medium; collagen-coated flasks; PDIOs mechanically split and cultured in Matrigel	HNE cells differentiated 21–28 days at ALI in PneumaCult-ALI medium; PDIOs pretreated ~20 h with modulators prior to FIS assay	Ciliated cells: Yes Goblet cells: Yes Basal cells: Yes Ionocytes: NR Barrier markers (ZO-1/E-cadherin): NR Functional validation: Electrophysiology	Not applicable	HNE cells: electrophysiology after 48 h pretreatment; PDIOs: 1 h FIS assay
**Ramezanpour M, 2026** [31]	Primary human nasal epithelial cells (HNECs); comparison of: monolayer, ALI culture, Dome organoids (3D), ALI organoids (3D apical-out)	non-CRS controls (4): 3 males and 1 female, 26–52 yrs CRSwNP patients (4): 3 males and 1 female, 37–64 yrs; one with asthma	Nasal brushings from middle turbinate (controls: septoplasty/skull base surgery; CRSwNP: nasal polyp surface)	Cells expanded as monolayers → embedded in Matrigel. ∘ Dome organoids: 50 µL Matrigel + 50 µL Airway Organoid Seeding Medium with 0.4 × 10^4^ cells, 24-well suspension plates. ∘ ALI organoids: 100% Matrigel-coated Transwell; 300,000 cells/mL in 5% Matrigel seeded apically.	Airway Organoid Differentiation Medium; medium changed 3×/week. Differentiation duration: 14 days. ALI monolayer differentiation: PneumaCult-ALI differentiation medium for 14 days.	Ciliated cells: Yes Goblet cells: Yes Basal cells: Yes Ionocytes: NR Barrier markers: NR Functional validation: CBF	Not applicable	Monolayer collected at day 7 (confluence). ALI and organoids collected after ~3 weeks (fully differentiated). Ciliary beat frequency measured at 45 days.
**Alosio GM, 2025** [32]	HNO-ALI	Healthy donors (2): female, 25–50 years	Nasal washes and swabs	Enzymatic digestion (airway organoid medium + Collagenase + Amphotericin B) → Debris removal and cell pelleting → Embedding in Matrigel^®^ → 3D expansion for 5–7 days	Organoids dissociated into single cells → Seeded on Transwell^®^ inserts (3 × 10^5^ cells/well) → Initial culture in airway organoid medium + EGF + Y-27632 → ALI induction (After 4 days) → Basolateral PneumaCult-ALI medium^®^ → Apical air exposure (37 °C, 5% CO_2_) → Media change every 4–5 days → Total differentiation time: 21 days	Ciliated cells: Yes Goblet cells: Yes Basal cells: Yes Ionocytes: NR Barrier markers: NR Functional validation: CBF	0.01	1, 3, and 6 days post-infection
**Boyd AI, 2025** [33]	HNO-ALI	Adult donors; multiple independent HNO lines; long-term expandable stem-cell-derived lines	Nasal wash + midturbinate swab; processed on ice until centrifugation	Tissue-resident stem cells propagated ex vivo as 3D organoids; dispersed and plated as monolayers on Transwells; differentiated at ALI	Differentiated at ALI to form mucociliary epithelium; experiments performed at 34 °C to mimic nasal physiology	Ciliated cells: Yes Goblet cells: Yes Basal cells: Yes Ionocytes: NR Barrier markers: NR Functional validation: None (infection readouts only)	Not expressed as MOI (bacterial colonization model; CFU-based inoculum)	24 h and 48 h colonization
**Wan Z, 2025** [34]	3D patient-derived airway organoids	Healthy donors (N/A)	Noninvasively from nasal mucosa	N/A	Two-phase system (expansion + differentiation); maturation into multicellular nasal epithelium; 96-transwell monolayer format	Ciliated cells: Yes Goblet cells: Yes Basal cells: Yes Ionocytes: NR Barrier markers: NR Functional validation: None (neutralization assays)	Not applicable	N/A
**Zhu L, 2025** [35]	3D patient-derived airway organoids	Pediatric donors (4): 5–12 years; suspected PCD, later excluded	Paired inferior turbinate nasal swab + bronchial tissue via bronchoscopy from same patients	Enzymatic digestion → Matrigel embedding → Defined airway organoid medium → Serial passaging	No ALI differentiation; 3D submerged Matrigel organoid culture	Ciliated cells: Yes Goblet cells: Yes Basal cells: Yes Ionocytes: NR Barrier markers: NR Functional validation: CBF	N/A	24 h and 48 h post-RSV infection
**Aloisio GM, 2024** [36]	HNO-ALI	Infant-derived nasal epithelial stem cells (4); compared with older donor-derived HNO-ALIs (4)	Nasal washes and swabs	Enzymatic digestion in airway organoid medium + collagenase + amphotericin B → Cell pelleting and debris removal → Embedding in Matrigel^®^ → 3D expansion for 5–7 days in growth medium	Organoids dissociated into single-cell suspension → Seeded on Transwell^®^ inserts (3 × 10^5^ cells/well) → Initial culture in airway organoid medium + EGF + Y-27632 → After 4 days → ALI induction → Basolateral PneumaCult-ALI medium → Apical air exposure (37 °C, 5% CO_2_) → Media changed every 4–5 days → Total differentiation time: 21 days	Ciliated cells: Yes Goblet cells: Yes Basal cells: Yes Ionocytes: NR Barrier markers: NR Functional validation: CBF	Low MOI (as per standard HNO-ALI viral infection protocol)	Multiple post-infection timepoints (early and late infection phases)
**Li L, 2024** [37]	Primary HNE progenitor cell-derived 3D apical-out nasal organoids cultured in CAH acid hydrogel system	CRS patients undergoing surgery (N/A): 20 independent donor-derived organoid lines	Human nasal mucosa biopsies	Isolation of HNE cells via mechanical and enzymatic dissociation → Expansion in feeder-based culture system → Embedding in CAH hybrid hydrogel (collagen I-based) → Progressive 3D branching (day 5–10) → Transition to spheroid morphology under differentiation medium	Switch to differentiation medium after expansion phase → Differentiation timeline approx. day 11–24 Emergence of: ∘ Goblet cells (~day 17) ∘ Ciliated cells (~day 24) ∘ Formation of tight junctions (ZO-1+) ∘ Development of apical-out polarity via ECM degradation	Ciliated cells: Yes Goblet cells: Yes Basal cells: Yes Ionocytes: NR Barrier markers (ZO-1): Yes Functional validation: None	Not applicable	Differentiation monitored from day 5 to day 24. Functional differentiation endpoint ~day 24.
**Li C, 2023** [38]	3D nasal organoids (NsO), airway organoids, alveolar organoids; differentiated 3D organoids and organoid monolayers	Healthy donors (N/A)	Inferior turbinate nasal brushings using flocked swab	Adult stem cell-derived → 3D expansion → Passaged every 2–3 weeks → Ratio 1:3–1:10 → Maintained up to 6 months	Organoids dissociated and plated onto Transwell inserts; differentiated under ALI conditions for ~21 days	Ciliated cells: Yes Goblet cells: Yes Basal cells: Yes Ionocytes: NR Barrier markers: NR Functional validation: None (infection kinetics)	MOI 0.1 (alveolar organoids) MOI 1 (flow cytometry experiment)	2 h adsorption Replication assessed at indicated hours post-infection 24 h.p.i. in inhibitor experiments
**Amatngalim GD, 2022** [39]	HNOs derived from 2D differentiated HNE	CF patients (N/A)	Nasal brushing	Stepwise approach: 2D expansion of HNECs → 2D ALI differentiation → conversion into 3D airway organoids	HNECs expanded in collagen IV-coated plates with growth factors (FGF7, FGF10, EGF, HGF); differentiated in 2D ALI cultures on transwells; subsequently converted into 3D organoids; optimized culture with neuregulin-1β and IL-1β	Ciliated cells: Yes Goblet cells: Yes Basal cells: Yes Ionocytes: NR Barrier markers: NR Functional validation: FIS	Not applicable	Functional assay time-dependent CFTR swelling
**Chiu MC, 2022** [40]	HNO-ALI model	10 lines of human nasal organoids were established using freshly isolated nasal epithelial cells from 9 healthy donors	Nasal brushing	Basal epithelial stem cells expanded and embedded in Matrigel to form 3D HNOs	Organoids dissociated and plated onto Transwell inserts; differentiated under ALI conditions for ~21 days	Ciliated cells: Yes Goblet cells: Yes Basal cells: Yes Ionocytes: NR Barrier markers: NR Functional validation: CBF	0.1	24 h, 48 h, 72 h post-infection
**Liu Z, 2022** [41]	Primary patient-derived 3D HNE organoids (Matrigel-embedded)	CF and non-CF patients (N/A)	Bilateral nasal brushing	HNE cells expanded using CRC → Feeder layer system with irradiated fibroblasts → Cells embedded in Matrigel (≥9 mg/mL protein concentration) → 500 cells/µL suspension → 2500 cells per replicate well → Cultured in Ultroser-G medium → Media change every other day	Spontaneous differentiation in 3D Matrigel → Functional maturity by day 21	Ciliated cells: Yes Goblet cells: Yes Basal cells: Yes Ionocytes: Yes (FOXI1+) Barrier markers (ZO-1): Yes Functional validation: FIS	Not applicable	Lumen formation: day 3–7 Functional endpoint (baseline lumen analysis/imaging): day 21 Imaging and fixation: up to day 28–42
**Ramezanpour M, 2022** [42]	Primary patient-derived 3D HNE organoids (Matrigel-embedded)	CRS patients (6): 4 males, 2 females; age 30–73 years	Inferior turbinate nasal brushings from CRS patients undergoing endoscopic skull base surgery	HNECs expanded as monolayer → embedded in 40% Matrigel → seeded at 180,000 cells/mL (~50,000 cells/cm^2^) in airway organoid seeding medium	Airway organoid differentiation medium (STEMCELL Technologies); medium changed 3×/week; differentiation assessed up to 20 days	Ciliated cells: Yes Goblet cells: Yes Basal cells: Yes Ionocytes: NR Barrier markers (ZO-1, E-cadherin): Yes Functional validation: CBF	Not applicable	Morphology assessed from day 2 to day 20; differentiation markers measured up to day 20; ciliary function evident at ~4 weeks
**Rodenburg LW, 2022** [43]	Primary human nasal epithelial organoids derived from minimally invasive nasal brushings	CF patients (N/A)	Minimally invasive nasal brushings	Epithelial sheets from differentiated ALI cultures mechanically disrupted and embedded in matrix to generate 3D nasal organoids	Basal cells expanded → differentiated at ALI → epithelial sheets embedded to generate 3D nasal organoids	Ciliated cells: Yes Goblet cells: Yes Basal cells: Yes Ionocytes: NR Barrier markers: NR Functional validation: FIS	Not applicable	Screening: 3 h (384-well primary screen) Validation swelling assay: 120 min (AUC over 2 h)
**Anderson JD, 2021** [44]	HNE organoid Swelling Model	CF patients (18) non-CF controls (5)	Nasal brushing	3D spherical organoid culture; parallel 2D monolayer cultures for short-circuit current comparison	FIS assay performed using automated imaging; CFTR modulators tested (ivacaftor, tezacaftor, elexacaftor combinations)	Ciliated cells: NR Goblet cells: NR Basal cells: NR Ionocytes: NR Barrier markers: NR Functional validation: FIS, Electrophysiology	Not applicable	0–8 h forskolin-induced swelling assay (AUC analysis)
**Sette G, 2021** [45]	Patient-derived HNE CRC-expanded airway epithelial stem cells	CF patients (14): various genotypes Includes F508del homozygous and rare compound heterozygotes	Nasal brushing	CRC expansion with feeder layer + ROCK inhibitor Embedding in Matrigel	ALI transwell differentiation ~3–4 weeks → lumen-forming organoids with beating cilia	Ciliated cells: Yes Goblet cells: Yes Basal cells: Yes Ionocytes: NR Barrier markers: NR Functional validation: FIS, Fluid reabsorption	Not applicable	48 h drug exposure (biochemical assays) 3 weeks organoid differentiation 3–4 weeks ALI differentiation
**Liu Z, 2020** [10]	Patient-derived 3D HNE organoid model	Non-CF controls (12): age 16–40 years CF patients (36): age 1–51 years Genotypes included minimal function and residual function mutations	Bilateral nasal brushings	Cells expanded up to passage ≤ 3 Seeded in Matrigel (≥9 mg/mL protein concentration) 500 cells/µL suspension 2500 cells per replicate well Cultured in Ultroser-G medium Lumens visible by day 3–7 Functional evaluation performed at day 21 Fixation and analysis up to 28–42 days	Primary HNE cells expanded using CRC → Cells embedded in ≥9 mg/mL Matrigel → Seeded at 500 cells/µL (2500 cells per well) → Cultured in Ultroser-G medium → Media changed every other day → Differentiation occurred spontaneously in 3D culture → Functional maturity achieved by day 21	Ciliated cells: Yes Goblet cells: Yes Basal cells: Yes Ionocytes: Yes (FOXI1+) Barrier markers (ZO-1): Yes Functional validation: FIS, Electrophysiology, CBF	Not applicable	Functional assays performed at day 21, with extended FIS evaluation up to 8 h.

Abbreviations: ALI, air–liquid interface; CAH, collagen–alginate–hyaluronic acid hydrogel; CBF, ciliary beat frequency; CF, cystic fibrosis; CRC, conditional reprogramming culture; CRS, chronic rhinosinusitis; CRSwNP, chronic rhinosinusitis with nasal polyps; EGF, epidermal growth factor; FIS, forskolin-induced swelling; FOXI1, forkhead box I1; HNE, human nasal epithelium; HNECs, human nasal epithelial cells; HNO, human nasal organoid; HNO-ALI, human nasal organoid–air–liquid interface; MOI, multiplicity of infection; NsO, nasal organoids; NR, not reported; PCD, primary ciliary dyskinesia; PDIOs, patient-derived intestinal organoids; RSV, respiratory syncytial virus; WT, wild type; ZO-1, zonula occludens-1.

## Data Availability

The data that support the findings of this study are available from the corresponding author upon reasonable request.

## Supplementary Materials

The following supporting information can be downloaded at: https://www.mdpi.com/article/10.3390/jcm15114016/s1, Table S1: PRISMA Check list;

## Abbreviations

The following abbreviations are used in this manuscript:

ALI	Air–Liquid Interface
CF	Cystic Fibrosis
CFTR	Cystic Fibrosis Transmembrane Conductance Regulator
CRC	Conditional Reprogramming Culture
CRS	Chronic Rhinosinusitis
FIS	Forskolin-Induced Swelling
HNO-ALI	Human Nasal Organoid Air–Liquid Interface
hNECs	Human Nasal Epithelial Cells
MOI	Multiplicity of Infection
PRISMA	Preferred Reporting Items for Systematic Reviews and Meta-Analyses
QUIN	Quality In Non-randomized Studies
